# Therapeutic Effect of Bifidobacterium Administration on Experimental Autoimmune Myasthenia Gravis in Lewis Rats

**DOI:** 10.3389/fimmu.2019.02949

**Published:** 2019-12-19

**Authors:** Elena Rinaldi, Alessandra Consonni, Chiara Cordiglieri, Grazia Sacco, Camilla Crasà, Alessandra Fontana, Lorenzo Morelli, Marina Elli, Renato Mantegazza, Fulvio Baggi

**Affiliations:** ^1^Neurology IV – Neuroimmunology and Neuromuscular Diseases Unit, Fondazione IRCCS Istituto Neurologico Carlo Besta, Milan, Italy; ^2^Department for Sustainable Food Process, Università Cattolica del Sacro Cuore, Piacenza, Italy; ^3^AAT—Advanced Analytical Technologies, Fiorenzuola d'Arda, Italy

**Keywords:** MG, EAMG, probiotics, immunoregulation, therapeutic treatment

## Abstract

Beneficial effects of probiotics on gut microbiota homeostasis and inflammatory immune responses suggested the investigation of their potential clinical efficacy in experimental models of autoimmune diseases. Indeed, administration of two bifidobacteria and lactobacilli probiotic strains prevented disease manifestations in the Lewis rat model of Myasthenia Gravis (EAMG). Here, we demonstrate the clinical efficacy of therapeutic administration of vital bifidobacteria (i.e., from EAMG onset). The mechanisms involved in immunomodulation were investigated with *ex vivo* and *in vitro* experiments. Improvement of EAMG symptoms was associated to decreased anti-rat AChR antibody levels, and differential expression of TGFβ and FoxP3 immunoregulatory transcripts in draining lymph nodes and spleen of treated-EAMG rats. Exposure of rat bone marrow-derived dendritic cells to bifidobacteria or lactobacilli strains upregulated toll-like receptor 2 mRNA expression, a key molecule involved in bacterium recognition via lipotheicoic acid. Live imaging experiments of AChR-specific effector T cells, co-cultured with BMDCs pre-exposed to bifidobacteria, demonstrated increased percentages of motile effector T cells, suggesting a hindered formation of TCR-peptide-MHC complex. Composition of gut microbiota was studied by 16S rRNA gene sequencing, and α and β diversity were determined in probiotic treated EAMG rats, with altered ratios between Tenericutes and Verrucomicrobia (phylum level), and Ruminococcaceae and Lachnospiraceae (family level). Moreover, the relative abundance of Akkermansia genus was found increased compared to healthy and probiotic treated EAMG rats. In conclusion, our findings confirms that the administration of vital bifidobacteria at EAMG onset has beneficial effects on disease progression; this study further supports preclinical research in human MG to evaluate probiotic efficacy as supplementary therapy in MG.

## Introduction

Myasthenia Gravis (MG) is a chronic autoimmune disease characterized by the presence of serum autoantibodies against the nicotinic acetylcholine receptor (AChR) at the neuromuscular junction (NMJ) in a large proportion of patients; AChR-specific antibodies lead to the alteration and destruction of NMJ causing muscle weakness and fatigability, major clinical symptoms in MG (1). Experimental autoimmune myasthenia gravis (EAMG), induced in susceptible strains as the Lewis rat or the C57Bl/6 mouse, is a well-characterized model to study the mechanisms involved in MG and novel therapeutic treatments (2).

Over the past years, many studies have been conducted and demonstrated the benefits of probiotic treatment in animal models of inflammatory diseases, such as experimental arthritis (3), experimentally induced colitis (4) and experimental autoimmune encephalomyelitis (5). Effects of probiotic administration on clinical symptoms and on immune mechanisms of EAMG has been investigated by us and other groups, according to a preventive or prophylactic/preventive protocol (6, 7). We previously demonstrated the effects of the preventive administration of two bifidobacteria strains or two lactobacilli strains in the Lewis rats EAMG model. We observed that probiotics significantly attenuated EAMG symptoms, decreased serum anti-rat AChR antibody levels and increased muscle AChR content. Pro-inflammatory and immunoregulatory transcripts were found differentially expressed in primary and secondary immune organs, and increased levels of Transforming Growth Factor-β (TGFβ) were measured in EAMG rat serum (6).

Orally administered probiotics exert their function in the gut, interacting with epithelial cells and the gut associated lymphoid tissue (GALT). GALT defends the host from pathogenic microorganisms and it is influenced by intestinal microbiota that lives symbiotically in the human gut. Dendritic cells (DCs), IgA-producing B cells, T helper1 (Th1), T helper 17 (Th17), and T regulatory (Treg) cells are the main players of the mucosal firewall that protects gut from external threats (8). In particular, DCs keep the immune system on high alert and balance T cell responses to pathogenic and not to commensal bacteria (9). Indeed, DCs can receive antigens from CX3CR1^+^ macrophages and intestinal epithelial cells, and extend dendrites toward the gut lumen to capture bacteria (10, 11). Upon antigen uptake, DCs migrate to mesenteric lymph nodes (LNs) to interact with T cells and shape the intestinal immune response (12).

Probiotics exert beneficial effects on gut microbiota homeostasis, and can contribute in restoring the eubiosis condition. The most abundant taxa colonizing the human gut are *Firmicutes* and *Bacteroidetes* and an altered ratio between the abundance of these taxa can be considered an index of intestinal dysbiosis. Besides this effect, probiotics also modulate inflammatory immune responses and foster the immunological surveillance; in this regards, it has been demonstrated that certain Lactobacilli strains stimulate the gamma interferon (IFNγ) and tumor necrosis factor (TNF) production, key molecules involved in the maturation and proliferation of immune cells (13), *Lactobacillus casei* Shirota induces IL12 production and promotes T helper cells development (14), *Lactobacillus rhamnosus* GG induces CD4^+^CD25^+^Foxp3^+^ T cell expansion in mesenteric LNs (15), strains of *Bifidobacterium animalis* and *Bifidobacterium longum* are able to support Th1 response, whereas strains of *Bifidobacterium bifidum* induce Th17 polarization (16).

In this study, we report the clinical efficacy of the therapeutic bifidobacterium administration on EAMG course, and that vital bacteria are more potent compared with inactive (heat exposed) bacteria. Moreover, we showed probiotic interactions with immune cells in the gut (namely the Peyer's Patches), through *ex vivo* and *in vitro* immunofluorescence analyses, and that probiotic altered the motility patterns of AChR-specific effector Tcells when co-cultured with probiotic-exposed bone marrow DC (BMDC), by means of live imaging microscopy. Lastly, we investigated gut microbiota composition of probiotic-treated EAMG rats by NGS 16S rRNA analysis, showing greater α and β diversity during EAMG course.

## Materials and Methods

### Animals

Female Lewis rats, 6–8 weeks old, were purchased from Charles River Laboratories Italia (Calco, Italy) housed at the animal facility of the Foundation IRCCS Neurological Institute Carlo Besta. Rats were housed in groups of three in cages with artificial circadian 12-h light/12-h dark cycle, maintained at air-conditioned room with temperature of 23°C at all time, with free access to a standard stock diet and water provided *ad libitum*. Procedures involving animals were approved by the Institute Ethical Board and Italian Ministry of Health (1064/2015- PR) and were performed in respect to the Italian Principle of Laboratory Animal Care (D.Lgs 116/92 and D.Lgs 26/2014), in accordance to European Communities Council Directive 86/609/EEC and 2010/63/UE. Animals were sacrificed after deep anesthesia obtained by carbon dioxide; low-grade anesthesia with 2% isoflurane (60:40 N_2_O: O_2_, flow rate 0.8 l/min) was induced in animals prior to immunizations and treatments.

### TAChR Preparation

TAChR was purified from *Torpedo californica* electric organ tissue (Aquatic Research Consultants), according to (17). Briefly, the electric tissue was homogenized in 10 mM sodium phosphate buffer, 1 mM EDTA, 0.02% NaN_3_, 0.01 mM PMSF, pH 7.8 for 3 min, and then centrifuged for 1 h at 100,000 × g at 4°C. Pellet was resuspended in ice-cold water and the pH adjusted to 11.0 with NaOH; membranes were centrifuged for 30 min at 100,000 × g at 4°C. AChR-containing membranes were homogenized for 2 min and the receptor solubilized with 2% sodium deoxycholate, overnight at 4°C. The detergent was removed by progressive dialysis, and TAChR stored at −80°C. TAChR concentration was quantified by the standard radioimmunoprecipitation protocol with [^125^I]-α bungarotoxin (αBTX) (PerkinElmer), according to Lindstrom et al. (18). [^125^I]-αBTX in samples was determined by a gamma counter (PerkinElmer). To evaluate the aspecific binding, serum samples were pre-incubated with an excess of unlabelled αBTX and counts per minutes (cpm) were subtracted from test samples. The specific activity of TAChR preparation used to induce EAMG was 1.19 nmol/mg, expressed as the α-BTX binding sites/mg of total protein content (micro BCA assay).

### Experimental Autoimmune Myasthenia Gravis (EAMG) Model

Experimental MG model was induced according to a consensus protocol (2) by a single subcutaneous immunization in the hind limbs (multiple sites) with 50 μg of TAChR emulsified in Complete Freud Adjuvant (CFA; Difco) supplemented with 1 mg/rat of *Mycobacterium tubercolosis* (total volume 200 μl). Each animal was weighed and scored at the beginning of the experiment, and at least twice weekly until the end of the experiment; clinical scores were taken every 24 h or less if the animals demonstrated severe weakness (2). EAMG clinical score was assessed by researchers, blinded to animal treatment, after 30 s exercise, using a manual grip strength test. Disease severity was graded as follows: grade 0, normal strength; grade 1, mildly decreased activity and weak grip or cry; grade 2, clinical signs present before exercise (tremor, head down, hunched posture, weak grip); grade 3, severe clinical signs at rest, no grip; grade 4, sacrifice, humane endpoint. EAMG was confirmed by Piridostigmine test (i.p. injection). The experiments were concluded 8 weeks post TAChR/CFA immunization.

### Generation of R97-116 Teff Cell Line

Lewis rats were immunized with 200 μg of R97-116 peptide (CASLO), the immunogenic region of rat AChR α-subunit, in CFA. Draining lymph nodes were aseptically removed 10 days post immunization, and LNCs suspensions were stimulated with R97-116 (10 μg/ml) in complete RPMI-1640 medium (Euroclone), containing 1% Na-pyruvate, 1% non-essential amino acids, 1% L-glutamine, 1% penicillin-streptomycin, 50 μM 2-mercaptoethanol, 2% normal rat serum (19). Antigen specific Teff were maintained by restimulation with R97-116 peptide every 15 days, and expanded with IL2 (10 U/ml) every 3 days.

### Probiotic Strains and Treatment Protocols

*Lactobacillus crispatus* LMG P-23257 (LC), *Lactobacillus rhamnosus* ATCC 53103 (LR), *B. animalis* subsp. *lactis* BB12® (BA, from CHR Hansen, Denmark) and *B. animalis* subsp. *lactis* LMG S-28195 (BL) were used. All strains were grown at AAT laboratory; briefly, lactobacilli were cultured in De Man, Rogosa & Sharp (MRS) broth (Difco) at 37°C in microearophilic conditions for 18 h, and bifidobacteria were grown in MRS broth supplemented with 0.05% cysteine at 37°C by anaerobic incubation for 24–48 h. Enumeration of viable bacterial cells was performed on selective media (MRS for lactobacilli, and Transoligosaccharide propionate agar medium added with 50 μg/ml mupirocin for bifidobacteria) by decimal counts.

Bacterial cells were resuspended at 10^9^ CFU/150 μl in phosphate saline buffer (PBS), 20% glucose, 10% glycerol and stored at −80° C. Loss of bacterial viability was <2–4% over 2 months storage period.

Combinations of bifidobacteria (BBmix) or lactobacilli (LBmix) strains were orally administered at a cumulative dose of 2 × 10^9^ CFU/300 μl. Alternatively to vital bifidobacteria, EAMG rats were fed with heat exposed (90°C for 5 min) bifidobacteria (BBmix heat exposed). Twenty-two consecutive doses of probiotics were administered starting from disease onset (days 27–28); end of experiments was set at least 4 days after the last probiotic administration. Animals were randomly selected in experimental groups, and evaluation of EAMG symptoms was performed by researchers blinded to the treatment group allocation. Inguinal and popliteal lymph nodes, spleen, muscle and blood were collected at the end of the experiment.

### Anti-Rat AChR Antibodies in Serum

Anti-rat AChR antibodies were assayed in sera by radioimmunoprecipitation, according to Lindstrom et al. (18). Briefly, AChR was extracted from healthy rat muscle and labeled with 2 nM [^125^I]-αBTX. Sera were incubated overnight with [^125^I]-αBTX-rat AChR (0.5 pmol). Ab-AChR complexes were precipitated by adding an excess of rabbit anti-rat IgG (Sigma). Pellet was washed twice with cold 0.5% Triton X-100 (Carlo Erba) in PBS, and [^125^I]-αBTX labeled-rat AChR complexes in the pellet were evaluated by γ-counter (Perkin Elmer). The non-specific binding was subtracted from each sample. Anti-AChR antibodies titres were expressed as picomole of [^125^I]-αBTX binding sites precipitated per milliliter of serum (pmol/ml).

### Probiotic Interaction With GALT

To evaluate probiotic presence in the gut, bacteria were labeled with wheat germ agglutinin-Alexa Fluor 555 conjugate (WGA-AF555) (Thermo Fisher) for 10 min, followed by extensive washes. A dose of 10^9^ CFU bacteria was administered to Lewis rats, and gut samples were excised after 30–60 min, washed with PBS, fixed with paraformaldehyde (PFA, 4% in PBS) for 24 h and then transferred in sucrose (30% in PBS) for cryopreservation. Samples were included in Killik (Bio-Optica) and kept at −80°C, pending analysis. Serial 10 μm thick cryosections were stained with Hematoxilin and Eosin (images digitalized with ScanScope, Aperio technologies) or with the following antibodies: mouse anti-vimentin mAb (V9, Dako), mouse anti-cytokeratin mAb (MNF116, Dako), mouse anti-CD11c mAb (8A2; ThermoFisher), mouse anti-CD3 mAb (G4.18, eBioscience), followed by species-specific Alexa Fluor 488-conjugated secondary antibodies. Isotype control stainings were routinely performed in the immunofluorescence procedures. Nuclei were stained with DAPI (Thermo Fisher Scientific). Single plan and z-scan images were captured via confocal microscopy and Structured Illumination microscopy (SIM), using a 100X APO-TIRF (NA 1.49) objective, with 3D optical sectioning. Images were processed with Fiji software (20).

### Bone Marrow Dendritic Cells Cultures and Probiotic Interaction

Single cell suspensions of myeloid precursor cells were derived from bone marrows of femur and tibia of naïve Lewis rats and cultured in RPMI-1640 medium supplemented with 1% Na-pyruvate, 1% non-essential aminoacids, 1% penicillin-streptomycin, 1% L-glutamine, 50 μM 2-mercaptoethanol, 10% fetal bovine serum (complete RPMI-1640 medium), in presence of GM-CSF and IL4 (each at 20 ng/ml; Peprotech) for 7 days to induce differentiation into immature bone marrow dendritic cells (BMDCs). BMDCs were seeded into 8-well chamber slides (1 × 10^5^ cells/chamber) for 16 h and then WGA-AF555-labeled probiotics (1 × 10^7^ CFU/1 × 10^5^ BMDCs) were added for 4 h. BMDCs cultures were then washed and fixed with 4% PFA for 10 min. BMDCs were stained with anti-CD11c mAb (8A2; Thermofisher). The interaction between BMDCs and bacteria has been visualized with rabbit anti-TLR2 pAb (AbClonal) and mouse anti-LTA mAb (3811; GeneTex), followed by species-specific Alexa Fluor-conjugated secondary antibodies.

### Live Imaging Assay

BMDCs (2 × 10^6^ cells) were exposed to probiotics (2 × 10^8^ CFU) or TGFβ (10 ng/ml) for 4 h, extensively washed with PBS, and cultured with complete RPMI-1640 medium or with R97-116 peptide (10 μg/ml in complete RPMI-1640) for further 2 h. BMDCs were detached, counted and seeded (5 × 10^5^ cells/dish) on glass-inserted imaging collagen-coated dishes for 16 h. Then, freshly stimulated R97-116 specific CD4^+^Tcell lines, labeled with 5 μM CFSE for 10 min at 37°C, were added to BMDC cultures (1.5 × 10^6^ cells/dish).

Time-lapse video microscopy was performed using a live-imaging Nikon set-up equipped with temperature/CO_2_ control unit (OKO lab). Differential interference contrast (DIC) and green channel images were acquired on a 512 × 512 pixel field of view, with 1.31 μm/pixel conversion. One hour recordings were performed with 30 s time-lapse interval using an inverted microscope (20X, 0.5 NA objective) and a Q-imaging Fast Camera (Roper scientific) and processed by NIS Elements AR software v3.1 (Nikon). TrackMate plug-in of Fiji software was used to automatically track T cells (21). Cells were defined as stationary if their path length was shorter than 10 μm every 10 min recording, or else they were classified as motile (22, 23).

### RT-qPCR

cDNA was synthesized from total RNA (TRIzol, Thermo Fisher Scientific) using random hexamers, and reverse transcriptase (SuperScript VILO cDNA Synthesis Kit, Thermo Fisher Scientific). Real-time quantitative PCR (qRT-PCR) was performed using Assay-on Demand Gene Expression Products (Thermo Fisher Scientific) specific for: IFNγ (Rn00594078_m1), IL6 (Rn01410330_m1), FoxP3 (Rn01525092_m1), TGFβ (Rn00572010_m1), TLR1 (Rn04181452_s1), TLR2 (Rn02133647_s1), TLR6 (Rn02121288_s1), CHRNA (Rn01278033_m1), Rapsyn (Rn014886207_m1), LRP4 (Rn01486328_m1); β-actin (Rn01515681_m1) was used as housekeeping endogenous gene. Target mRNA expression was calculated as mean 2^−ΔCt^ × 100 value, in which ΔCt is the difference between target and housekeeping Ct. Real-time PCR reactions were performed in duplicates using Viia7 Real-Time PCR System, according to the manufacturer's instructions.

### Stool Collection and Nucleic Acid Extraction

Stool samples were collected at day 0 (before immunization), day 30 (at EAMG onset) and at the end of experiment. Stool samples collected from the animals housed in the same cage were pooled and dissolved in Stool Nucleic Acid Collection and Preservation Tubes (Norgen Biotek Corp.), pending analysis. Bacterial DNA extraction was performed using Stool DNA Isolation Kit (Norgen), according to the manufacturer's instructions. Briefly, 400 μl of stool samples were mixed with lysis buffer (1:1.5 v/v) and homogenized using a flat-bed vortex. The supernatant was collected and transferred to a DNAse-free microcentrifuge tube, centrifuged to pellet any cell debris and loaded onto a spin-column. The bound DNA was washed, eluted and stored at −20°C.

Samples were: healthy rats (HD rats), EAMG rats at disease onset (EAMG onset), vehicle treated EAMG rats (EAMG chronic), EAMG rats treated with vital BBmix (EAMG BBmix vital), EAMG rats treated with heat exposed BBmix (EAMG BBmix heat exposed). Five replicates were considered for HD rats and EAMG BBmix vital samples; four replicates for EAMG chronic samples; three replicates for EAMG onset and EAMG BBmix heat exposed.

### 16S rRNA NGS Sequencing

Purity and quantity of the bacterial DNA were confirmed by Bioanalyser 2100 (Agilent Technologies, USA) and NanoDrop 2000 (ThermoFisher) devices. Bacterial 16S rRNA variable regions (V2, V3, V4, V6, V8, V7-9) were amplified using specific primers (Metagenomic kit, Invitrogen) and completed by the addition of a PGM sequencing adaptor (P1) and unique barcode to allow multiplex analyses. Prior to NGS sequencing, quality and amplicon sizes were assessed using the Bioanalyser 2100. The samples were adjusted to a final concentration of 26 pM and attached to the surface of Ion Sphere particles (ISPs) according to the manufacturer's instructions. Manual enrichment of the resulting ISPs resulted in >95% templated- ISPs. Templated-ISPs were sequenced on either “314” (10 Mb.p.) or “316” (100 Mb.p.) micro-chips using the Ion Torrent Personal Genome Machine (Life Technologies, USA) for 850 flows.

### Metagenomic Analysis

Raw data from the Ion Torrent Personal Genome Machine were analyzed with the Ion Reporter™ Software 5.10 and the workflow Metagenomics 16S w1.1 to generate fastq sequences for the different 16S rRNA variable regions and a consensus sequence for each sample. Consensus fastq sequences were elaborated with QIIME 2 2018.8 (24). Specifically, demultiplexing and quality filtering were performed using the q2-demux plugin followed by denoising with DADA2 (25). Alpha-diversity metrics (observed OTUs, Shannon and evenness), beta-diversity metric (unweighted UniFrac) (26) and Principle Coordinate Analysis (PCoA) were estimated using q2-diversity after samples were rarefied (subsampled without replacement) to 9,805 sequences per sample. Taxonomy was assigned to OTUs using the q2-feature-classifier (27) classify-consensus-vsearch taxonomy classifier against the Greengenes 13_8 99% OTUs reference sequences (28). Hierarchical clustering using Pearson distances in MeV (29) was used to create a genus-level heatmap of the relative abundances.

### Statistical Analysis

Experimental data were analyzed via one-way ANOVA or two-way ANOVA for normally distributed values, followed by Dunnett's multiple comparison test or via Kruskal–Wallis test for not normally distributed values; normal distribution of data was evaluated via Kolmogorov test. All *p*-values were corrected for multiple comparisons. *P* < 0.05 was considered statistically significant. Graph Pad Prism was used for data elaboration and statistical analyses.

## Results

### Improvement of EAMG Symptoms After Therapeutic Treatment With Bifidobacteria

**Figure 1:**
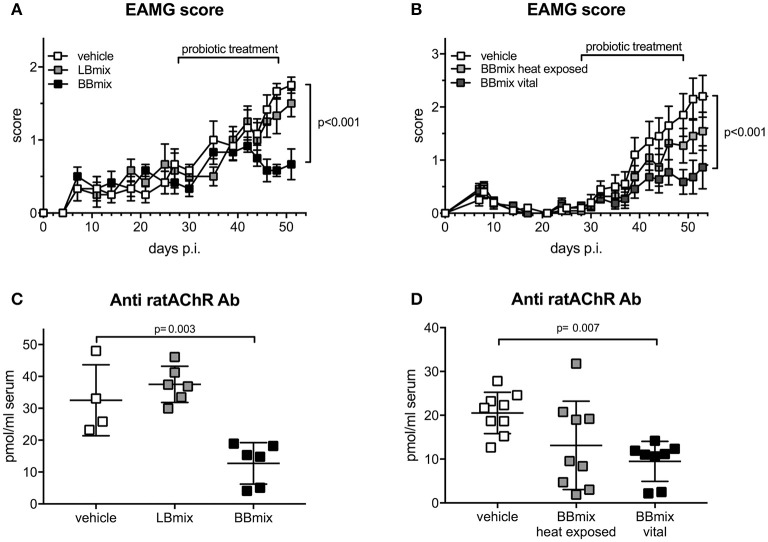
Therapeutic administration of vital bifidobacteria strains ameliorates EAMG course. **(A)** Clinical EAMG score (mean ± SEM) of EAMG animals treated with vehicle, LBmix or BBmix (*n* = 6 rats/group). **(B)** Clinical EAMG score (mean ± SEM) of EAMG animals treated with vehicle, BBmix heat exposed or BBmix vital (*n* = 11 rats/group). **(C,D)** Anti-rat AChR Ab serum titer (pmol/ml of rat serum, mean ± SD) of treated-EAMG rats. Two-way ANOVA test with Tukey's *post-hoc* test for multiple-comparisons was used for clinical score. One-way ANOVA test with Dunnett's multiple comparison test was used for anti-rat AChR. Corrected *p*-values are reported.

The therapeutic effect of bifidobacteria (BBmix) and lactobacilli (LBmix) administration was evaluated in EAMG Lewis rats, compared to vehicle-fed animals (Figure 1A). Probiotic treatments started at disease onset and throughout the chronic phase. BBmix was more effective than LBmix in ameliorating the disease course (Figure 1A; vehicle-EAMG, mean score 1.75 ± 0.27, LBmix-EAMG, mean score 1.5 ± 0.45, BBmix-EAMG, mean score 0.67 ± 0.52, corrected *p* < 0.001). The observed EAMG improvement in BBmix treated animals was confirmed by the assessment of animal weights, compared to control (vehicle fed) EAMG. The mean weights (grams ± SD) at the end of experiments were: BBmix-EAMG, 228 ± 10; vehicle-EAMG, 189 ± 33; LBmix-EAMG, 218 ± 15 (Supplementary Figure 1A). Next, we investigated whether the beneficial effect of BBmix could be associated to vital or heat exposed probiotics; administration of vital bacteria was significantly associated with a decreased clinical score, whereas heat exposed bifidobacteria did not modify the disease course (Figure 1B; vehicle-EAMG, mean score 2.2 ± 1.25, BBmix vital- EAMG, mean score 0.86 ± 1.34, corrected *p* < 0.001; BBmix heat exposed- EAMG, mean score 1.54 ± 1.19, corrected *p*-value = 0.26 vs. vehicle-EAMG). Again, EAMG improvement was paralleled by improvement of mean animal weight (Supplementary Figure 1B, BBmix vital- EAMG, grams 220 ± 25, vehicle-EAMG, grams 182 ± 37, BBmix heat exposed- EAMG, grams 193 ± 30).

Improvement of EAMG symptoms were confirmed by a significant reduction of serum antibody level against rat AChR in BBmix-EAMG rats (mean titer 12.73 ± 6.50 pmol/ml) compared to vehicle-fed (mean titer 32.53 ± 11.13 pmol/ml) or LBmix-EAMG rats (mean titer = 37.51 ± 5.67 pmol/ml) (Figure 1C). Similarly, anti-rat AChR antibody levels were found reduced in EAMG rats treated with vital bifidobacteria but not in heat exposed BBmix-fed rats (Figure 1D; BBmix vital-EAMG rats mean titer 9.49 ± 4.55 pmol/ml, vehicle-EAMG rats mean titer 20.53 ± 4.72 pmol/ml, corrected *p* = 0.007; BBmix heat exposed-EAMG mean titer = 13.13 ± 10.07 pmol/ml, corrected *p* = 0.06 ns). Clinical efficacy of BBmix treatment has been confirmed by RT-qPCR analysis for the expression of mRNAs encoding for CHRNA, Rapsyn and LRP4, key molecules of the NMJ involved in AChR stabilization on the postsynaptic membrane. Treatment of EAMG rats with vital BBmix is associated with decreased CHRNA1 and Rapsyn mRNAs, comparable to HD rats, whereas their expression was significantly upregulated in vehicle treated-EAMG animals (Supplementary Figure 2).

**Figure 2:**
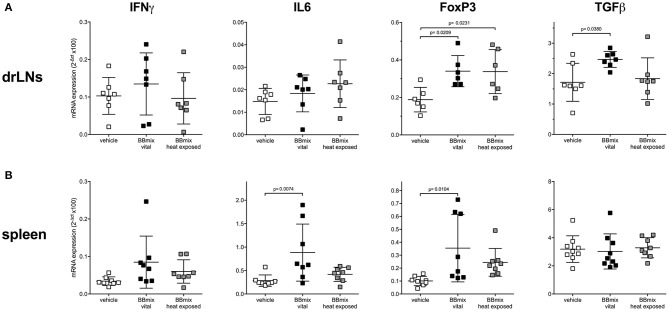
Differential expression of pro-inflammatory and regulatory transcripts in primary and secondary lymphoid organs of EAMG rats. qRT-PCR analysis of IFNγ, IL6, FoxP3, and TGFβ mRNAs (mean ± SD) in drLNs **(A)** and spleen **(B)** of EAMG rats treated with vehicle, BBmix vital or BBmix heat exposed. One-way ANOVA test with Dunnett's multiple comparison test was used to assess statistical significance. Corrected *p*-values are reported.

Since BBmix treatment, and especially BBmix vital, was associated with a significant EAMG improvement, we then studied by means of qRT-PCR the differential expression of IFNγ and IL6 (as proinflammatory markers) and of FoxP3 and TGFβ (as immunomodulatory markers) mRNA transcripts in draining lymph nodes (drLNs) and spleen isolated from BBmix treated EAMG rats (Figure 2). We did not detect differences for IFNγ in the different immunocompetent tissues analyzed, whereas IL6 mRNA was found increased in the spleen (corrected *p* = 0.0074). With regard to regulatory markers, we found increased expression of FoxP3 mRNA in drLNs (corrected *p* = 0.0209) and spleen (corrected *p* = 0.0104) of animals treated with BBmix vital; also TGFβ mRNA was found upregulated in drLNs (corrected *p* = 0.0380).

### Detection of Probiotic in the GALT

**Figure 3:**
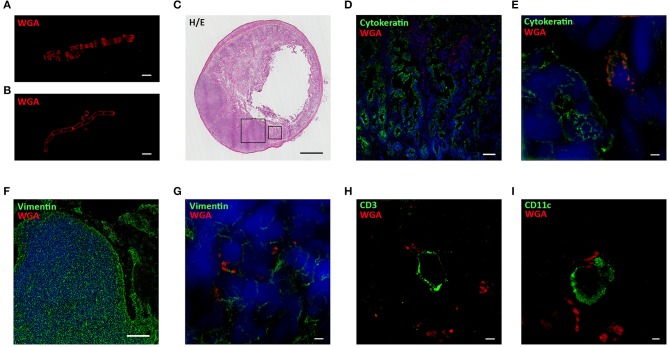
Probiotics interact with immune cells located in Peyer's Patches and villi. SIM images of WGA-AF555 (red) labeled-BA **(A)** and LR **(B)**. **(C)** Hematoxilin and Eosin staining of rat small intestine. **(D)** Confocal and **(E)** SIM images of villi stained for cytokeratin (green) and nuclei (blue). WGA-AF555 LR (red) are located inside villi (confocal image scale bar: 50 μm). **(F)** Confocal and **(G)** SIM images of PP stained for vimentin (green) and nuclei (blue). WGA-AF555 LR (red) are located inside the PP (confocal image scale bar: 100 μm). SIM images of CD3^+^ cell (green) **(H)** and CD11c^+^ cell (green) **(I)**. WGA-AF555 LR (red) are detected nearby CD3^+^ and CD11c^+^ cells. SIM image scale bar: 2 μm.

Probiotic interaction with the host immune system occurs in the GALT and, among the lymphoid structures located in the intestinal mucosa, Peyer's Patches (PPs) are one of the main lymphocyte priming sites in response to microbial stimulation (30). To investigate the interaction of our probiotic strains within the GALT, bifidobacteria and lactobacilli were stained with WGA-AF555, a fluorescently labeled lectin that binds specifically to polar polysaccharides (31, 32) of bacterium wall (representative images of labeled BA, Figure 3A, and LR, Figure 3B). Lewis rats received a single dose (1 × 10^9^ CFU) of WGA-AF555 labeled probiotic and, after 30–60 min, the small intestine was removed and processed for histological (Figure 3C, H&E staining) and immunofluorescence analyses (Figures 3D–I). Fluorescently labeled bacteria were found localized inside intestinal villi (counterstained with a green-fluorescent mAb anti-cytokeratin) with confocal microscopy (Figure 3D) and SIM (Figure 3E), and within the PPs (counterstained with a green-fluorescent mAb anti-vimentin) (Figure 3F, confocal microscopy, and Figure 3G, SIM).

It is known that PPs are constituted by three regions containing different cell types: the follicular and interfollicular areas with a germinal center of proliferating B-lymphocytes, follicular dendritic cells and macrophages, and the corona or subepithelial dome, surrounding the follicle, populated by B-cells, T-cells, macrophages, and dendritic cells (33). In order to identify the main cell types within the PPs substructures proximal to WGA-AF555 labeled probiotic, serial sections of the small intestine were stained with a AF488-conjugated anti-CD3 to detect T lymphocytes and with a AF488-conjugated anti-CD11c to detect dendritic cells; super resolution analysis confirmed that WGA-AF555 bacteria were nearby CD3^+^ lymphocytes (Figure 3I) and CD11c^+^ dendritic cells (Figure 3H).

### Exposure of BMDCs to Probiotic Affects R97-116 Tcells Motility

**Figure 4:**
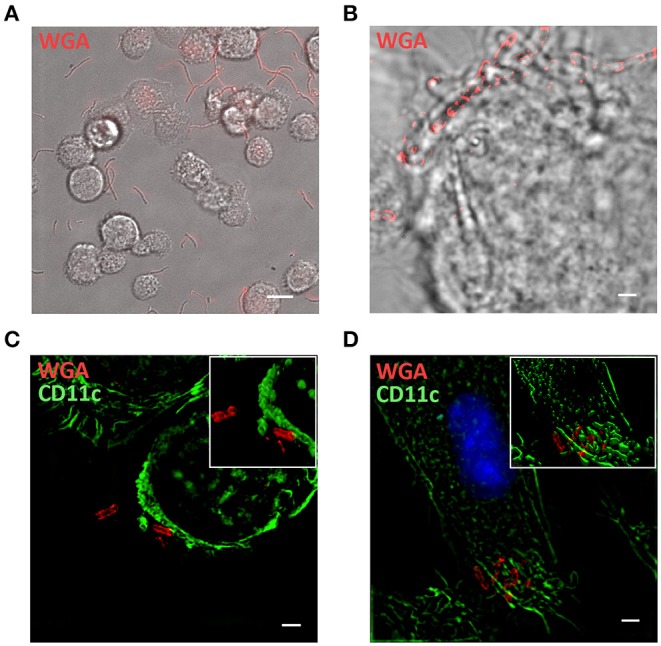
*In vitro* interaction between BMDCs and probiotics. **(A)** Single frame of BMDCs co-cultured with WGA-AF555 labeled-LR (red). Scale bar 20 μm. **(B)** SIM image of a BMDC physically interacting with a chain of WGA-AF555 labeled-LR (red). **(C,D)** SIM images of CD11c^+^BMDC (green) and WGA-AF555-LC and WGA-AF555-BA (red) respectively. Site of contact between BMDCs and bacterial cells are reported in insets (3D volume). SIM image scale bar: 2 μm.

The interaction between probiotics and immune cells was further investigated by *in vitro* experiments with rat BMDCs and T lymphocytes specific for the immunodominant peptide R97-116 of the rat AChR alpha subunit (34). BMDCs were exposed to WGA-AF555 labeled-probiotics for 60 min, and cultures were analyzed via live imaging microscopy (Figure 4A—single frame—and Supplementary Video 1). Most BMDCs are characterized by short movements to sample the surrounding microenvironment, while WGA-AF555-probiotics are either stably captured by BMDCs or free to move. Parallel cultures were processed for immunofluorescence analysis by SIM (Figures 4B–D), further confirming that single BMDCs (bright field) make multiple contacts with WGA-AF555 labeled-LR bacteria, arranged in chain (Figure 4B). Representative SIM images of CD11c^+^ BMDCs contacting WGA-AF555 labeled-probiotics are reported in Figure 4C (LC strain, single plan) and Figure 4D (BA strain, single plan), and in the reconstructed 3D images (insets Figures 4C,D), showing probiotics contacting BMDC plasma membrane.

**Figure 5:**
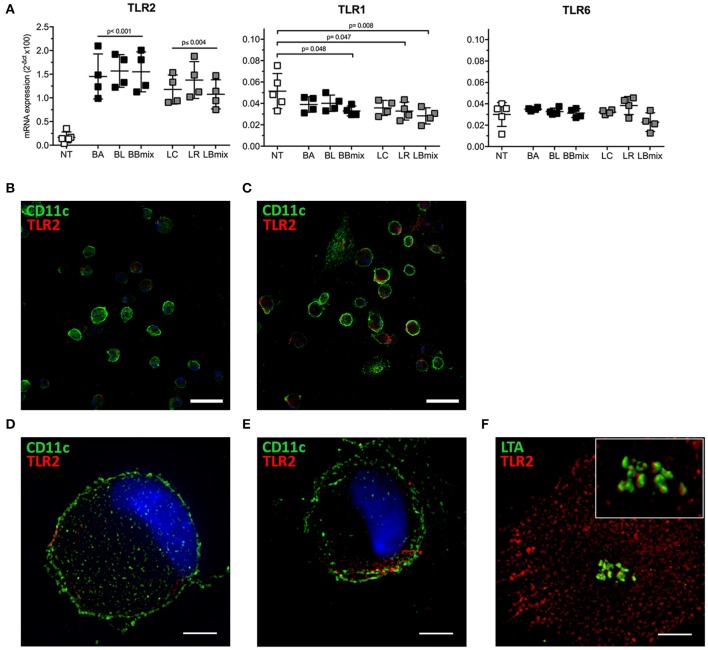
Differential expression of TLRs in BMDCs exposed to probiotics. **(A)** qRT-PCR analysis of TLR2, TLR1, and TLR6 in BMDCs exposed to single strains (BA, BL, LC, LR) and combinations (BBmix, LBmix) of probiotics for 4 h (mean ± SD). **(B)** Confocal and **(D)** SIM images of untreated CD11c^+^ cells (green) and TLR2 (red). **(C)** Confocal and **(E)** SIM images of LR-exposed CD11c^+^ cells (green) and TLR2 (red). **(F)** SIM image of LTA (green) and TLR2 (red) of BMDCs exposed to LC (inset: 3D volume). Statistical significance was assessed by one-way ANOVA test with Dunnett's multiple comparison test. Corrected *p*-values are reported. Confocal image scale bar: 50 μm; SIM image scale bar: 5 μm.

Toll-like receptors (TLRs) recognize multiple pathogens, including bacteria, viruses, fungi, and parasites, and their expression is regulated in both a cell type- and stimulus-dependent fashion (35). Hence, the differential expression of selected TLRs mRNA was evaluated in BMDCs exposed to bifidobacteria (BA, BL, BBmix) and lactobacilli (LC, LR, LBmix) (Figure 5). Increased TLR2 mRNA expression was observed in bifidobacteria- (*p* < 0.001) or lactobacilli-treated BMDCs (*p* ≤ 0.004) vs. untreated BMDCs; TLR1 transcript were downregulated (BBmix, *p* = 0.048; LR, *p* = 0.047; LBmix, *p* = 0.008) whereas TLR6 expression was not altered in any experimental condition. The increased expression of TLR2 transcript was confirmed via immunofluorescence analysis (Figures 5B–E). Confocal and super resolution microscopy studies demonstrated increased expression of TLR2 on the membrane of probiotic exposed-CD11c^+^ cells (Figures 5C,E) compared to untreated BMDCs (Figures 5B,D). Since TLR2 forms clusters in response to Lipoteichoic Acid (LTA), a component of the wall of Gram-positive bacteria (36), we investigated whether TLR2 upregulation observed on BMDCs could be associated with LTA, expressed on our probiotic strains, by means of SIM (Figure 5F). The analysis showed that LTA molecules (in green) are arranged in clusters and localized in proximity of TLR2 (in red) expressed by BMDCs, or even in close contact with TLR2 molecules (reconstructed 3D volume, Figure 5F inset).

**Figure 6:**
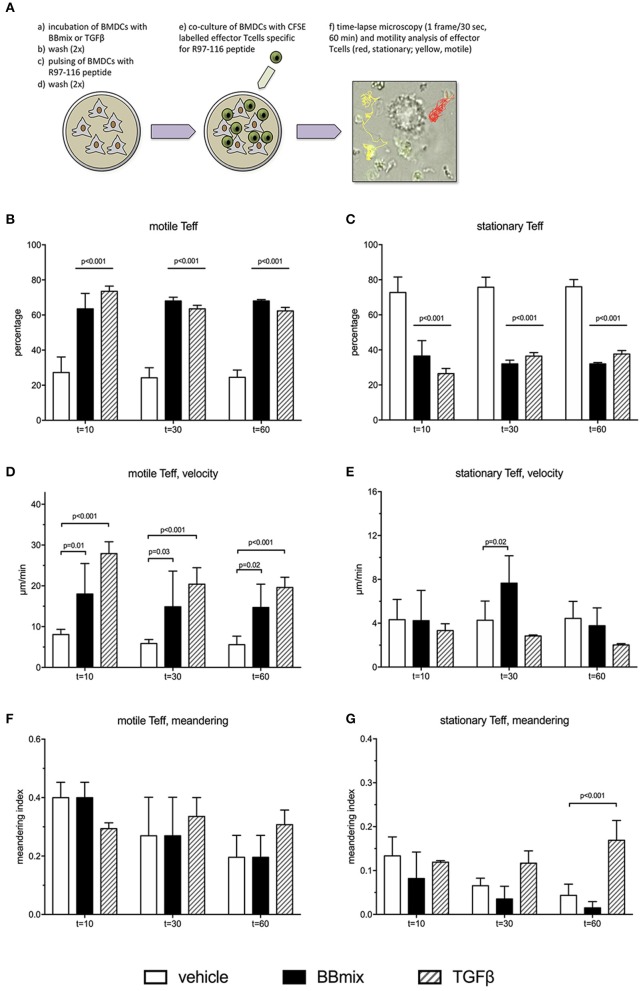
Treatment of BMDCs with probiotics modifies R97-116 T cell motility. **(A)** Experimental set-up of live imaging analyses of CFSE-labeled R97-116-specific T cells co-cultured with R97-116-loaded BMDCs, and example of motility analysis. **(B)** Percentage, **(D)** velocity and **(F)** meandering index of motile Teff. **(C)** Percentage, **(E)** velocity, and **(G)** meandering index of stationary Teff. Experimental conditions were: control cultures of BMDC, loaded with R97-116 peptide (empty bars), BMDC exposed to BBmix prior to antigen loading (black bars), BMDC exposed to TGFβ prior to antigen loading (striped bars). Statistical significance was assessed by two-way ANOVA test with Dunnett's multiple comparison test. Corrected *p*-values are reported.

To further evaluate the events associated with the probiotic-induced BMDCs immunomodulatory profile, rat BMDCs, grown in complete RPMI medium (as control) or exposed to BBmix or TGFβ, were subsequently loaded with peptide R97-116 and *in vitro* co-cultured with R97-116 specific CD4^+^ effector T cells (Teff) (Figure 6A). By means of live imaging microscopy, the motility pattern of Teffs was recorded (1 frame/30 s, 60 min recording) and analyzed with Fiji software, plug-in TrackMate (21). Representative movies are included as Supplementary Videos 2, 3. Cells were defined as stationary if their path length was shorter than 10 μm every 10 min recording, or else they were classified as motile (22, 23).

When antigen-specific Teff cells were co-cultured with control BMDCs (peptide-loaded but not exposed to BBmix), 27.3 ± 8.6% of tracked cells (total number of tracked cells = 300; 4 replicates) showed a motile pattern at *t* = 10 min (Figure 6B, empty bar) and, conversely, 72.7 ± 8.6% had a stationary pattern (Figure 6C, empty bar); percentages of motile Teff (as well as stationary Teff) did not change in the subsequent time points (stationary Teff: 75.7 ± 5.5% at *t* = 30 min and 75.3 ± 4.3% at *t* = 60 min). Exposure of BMDCs to BBmix resulted in an altered Teff motility pattern: 63.7 ± 9.0% were classified as motile at *t* = 10 min (*p* < 0.001 vs. control BMDCs, Figure 6B, black bar), and this percentage was stable at *t* = 30 and *t* = 60.

Then, mean velocity (expressed as μm/minute) and meandering index were calculated for either motile (Figures 6D,F) and stationary Teffs (Figures 6E,G): as expected, motile Teff cells had a mean velocity greater than stationary cells (8.1 ± 1.3 μm/min vs. 4.3 ± 1.9, Figures 6D,E respectively, empty bars) at *t* = 10, *t* = 30 and *t* = 60. Interestingly, when BBmix-exposed BMDCs were evaluated, mean velocity of motile Teff significantly increased compared with control BMDCs (*t* = 10, 18.2 ± 12.4 μm/min vs. 8.1 ± 1.3, corrected *p* = 0.01, Figure 6D, black bars), and this observation was confirmed at the subsequent time points. On the contrary, mean velocity of stationary Teffs was significantly different at *t* = 30 only (Figure 6E, black bars), and at *t* = 60 returned similar to *t* = 10.

Meandering index is a measure of the Teff cells patrolling while moving nearby BMDCs, that reflects the intrinsic difference between motile Teff cells, with a meandering index 0.4 ± 0.1 (*t* = 10, Figure 6F, empty bar) and stationary Teff cells (meandering index 0.13 ± 0.04, *t* = 10, Figure 6G, empty bar). No differences were observed when Teffs were co-cultured with BBmix-exposed BMDCs (Figures 6F,G, black bars).

In a previous study, we have reported increased expression of TGFβ in tissue culture supernatants from probiotic-treated BMDCs and in the serum from probiotic-treated EAMG rats, suggesting an immunomodulatory role for this pleiotropic cytokine. Hence, we exposed BMDCs to TGFβ (10 ng/ml) prior to antigen pulsing and co-culture with Teff cells (representative movie is reported as Supplementary Video 4). Interestingly, the percentage of tracked cells (total number of tracked cells = 225; 3 replicates) classified as motile (Figure 6B, striped bars) was significantly increased compared to Teff cells co-cultured with control BMDCs (corrected *p* < 0.001, Figure 6B, empty bars) and similar to what observed with BBmix-exposed BMDCs (Figure 6B, black bars), at *t* = 10, *t* = 30 and *t* = 60 time points. Again, the mean velocity of motile Teff cells was found increased (31.2 ± 4.9 μm/min, *t* = 10, Figure 6D, striped bars) compared to control co-cultures (corrected *p* < 0.001), a behavior similar to that observed in co-cultures with BBmix-exposed BMDCs (Figure 6D, black bars).

### Probiotics Influence Gut Microbiota in EAMG

**Figure 7:**
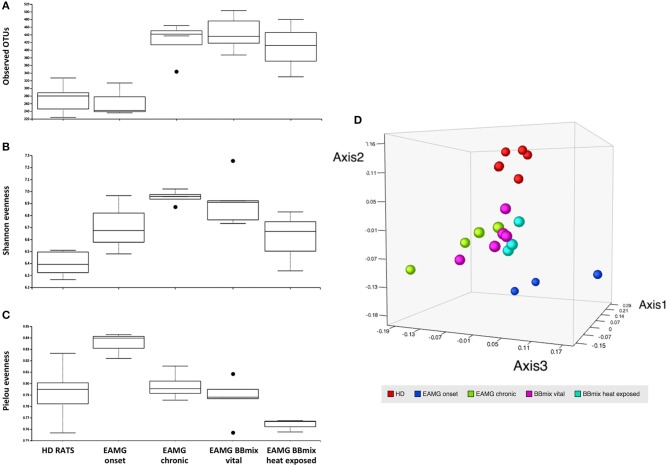
Treatment with Bifidobacteria modifies alpha and beta diversity of EAMG gut microbiota. **(A)** Observed OTUs, **(B)** Shannon index, **(C)** evenness, and **(D)** unweighted UniFrac distance PCoA of healthy rats (HD rats; red dots), EAMG rats 30 days after the induction of the disease (EAMG onset; blue dots), EAMG rats in the chronic phase (EAMG chronic; green dots), EAMG rats treated with vital bifidobacteria (EAMG BBmix vital; pink dots), and EAMG rats treated with heat exposed bifidobacteria (EAMG BBmix heat exposed; turquoise dots).

NGS analysis of gut microbiota was performed on stools collected from experimental animals at different time points: HD animals (day 0, before TAChR/CFA immunization), EAMG onset (at day 27, before treatments) and at the end of experiment, for each treatment groups (vehicle, BBmix and BBmix heat exposed). Stools collected from the animals housed in the same cage were pooled before processing for DNA extraction. Raw data from NGS sequencing were analyzed with the Ion Reporter™ Software 5.10 and the workflow Metagenomics 16S w1.1; consensus fastq sequences were then elaborated with QIIME 2 microbiome bioinformatics platform (version 2018.8). A total number of 1,255,155 reads were obtained, with an average of 59,769 reads per sample, and 934 OTUs identified. Alpha and beta diversity analyses were performed on the OTU table. The observed OTUs plot (Figure 7A) showed a similar number of OTUs in HD and EAMG onset groups, different from the OTU numbers in EAMG chronic, EAMG BBmix vital and EAMG BBmix heat exposed groups. The Shannon evenness analysis (Figure 7B) showed a more complex degree of α-diversity occurring across experimental groups, indicating a higher OTUs richness of gut microbiota in chronic, vehicle fed, EAMG animals, with a tendency to α-diversity reduction in BBmix heat exposed group. Concerning the Pielou's index for evenness, the highest value was observed in EAMG onset group (Figure 7C), pointing out that the microbiota species in these animals were more equally distributed compared to the other groups. β-diversity was evaluated by the unweighted UniFracPCoA analysis (Figure 7D), which allowed the identification of three separated clusters: HD (red dots), EAMG onset (blue dots) and a more heterogeneous cluster comprising EAMG chronic (green dots), EAMG BBmix vital (pink dots) and heat exposed (turquoise dots). Of note, HD, EAMG onset and EAMG chronic groups were quite separated, indicative of a greater degree of β-diversity, while it was possible to discriminate only a modest difference between probiotic treated EAMG rats.

**Figure 8:**
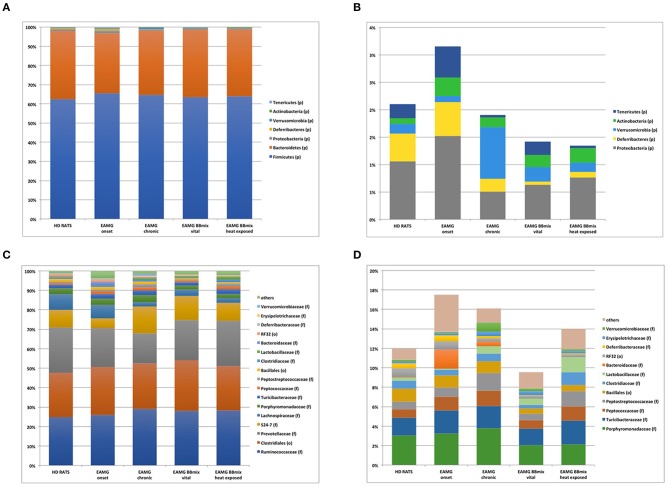
Bifidobacteria reduce the dysbiosis induced by the disease. Stacked barplot representing the taxonomic assignments at *phylum* level (phyla with relative abundance higher than 0.1%). Major phyla, defining on average the 98% of the bacterial community **(A)**, and minor phyla **(B)**. Stacked barplot representing the taxonomic assignments at *family* level (families with relative abundance higher than 1%). Dominant families, defining on average the 86% of the bacterial community **(C)**, and less abundant families **(D)**.

At taxonomical level, *Firmicutes* (relative abundance 62–64%) and *Bacteroidetes* (relative abundance 28–34%) were the dominant phyla in all groups (Figure 8A), together representing ~98% of the gut microbiota. *Firmicutes*/*Bacteroidetes* ratio (F/B, Table 1) did not show any difference across experimental groups. The analysis restricted to the phyla with relative abundance below 5% showed 5 main phyla: *Proteobacteria, Deferribacteres, Verrucomicrobia, Actinobacteria* and *Tenericutes* (Figure 8B). Within this subset, the *Tenericutes*/*Verrucomicrobia* ratio (T/V) was found significantly altered in EAMG onset group, compared to HD (T/V ratio 5.18 ± 1.3 vs. 2.21 ± 1.8, corrected *p* = 0.007), followed by a sharp decrease (0.1 ± 0.1) in chronic, vehicle fed, EAMG rats. Interestingly, treatment with vital probiotic partly restored the T/V ratio (0.96 ± 0.3), indicating a possible effect of the BBmix treatment on these phyla, not observed in heat exposed treated EAMG animals. At family level, *Ruminococcaceae* (relative abundance 27 ± 2%), *Prevotellaceae* (relative abundance 20 ± 3%) and an unclassified family belonging to the *Clostridiales* order (relative abundance 24 ± 1%) represented ~70% of the gut microbiota (Figure 8C), without differences. Major changes were observed in the less abundant families (relative abundance lower than 15%) (Figure 8D), showing a strong reduction of *Lachnospiraceae* in EAMG chronic group, and an increase of the *Ruminococcaceae*/*Lachnospiraceae* (R/L) ratio (EAMG chronic R/L ratio 13.22 ± 2.4; Table 1). Interestingly, probiotic treatment was associated with a decreased R/L ratio (EAMG BBmix vital R/L ratio 8.49 ± 0.8). A similar trend was showed by the relative abundances for *Peptoptococcaceae* and *Peptostreptococcaceae* families (Figure 8D), increased in EAMG chronic, unchanged in heat exposed BBmix, but reduced in vital BBmix group to values similar to HD rats (Figure 8D). The relative abundance heatmap (Supplementary Figure 3) showed a specific cluster in the EAMG onset group comprising the genera *Sutterella, Phascolarctobacterium, Dialister, Odoribacter, Lachnospira*, two unclassified genera belonging to *Rikenellaceae* and *Barnesiellaceae* families and one unclassified genus belonging to the *Bacteroidales* order. On note, the increase of *Akkermansia* in the EAMG chronic group, and the *Turicibacter* genus, reduced in vital BBmix group similar to what observed at the family level. Of interest, the relative abundance of *Lactobacillus* genus was also affected by vital bifidobacteria treatment and not by inactivated probiotic strains (Supplementary Figure 3).

**Table 1:** *Firmicutes/Bacteroidetes* (F/B), *Tenericutes*/*Verrucomicrobia* (T/V), and *Ruminococcaceae*/*Lachnospiraceae* (R/L) ratios in samples.

	**F/B ratio (phylum)**	**T/V ratio (phylum)**	**R/L ratio (family)**
**HD**	1.79 ± 0.3	2.21 ± 1.8	3.11 ± 0.5
**EAMG onset**	2.21 ± 0.7	5.18 ± 1.3^a^	3.93 ± 1.4
**EAMG chronic**	1.98 ± 0.5	0.10 ± 0.1^b^	13.22 ± 2.4^c^
**EAMG BBmix vital**	1.83 ± 0.3	0.96 ± 0.3	8.49 ± 0.8^d^^,^ ^f^^,^ ^g^
**EAMG BBmix heat exposed**	1.88 ± 0.4	0.37 ± 0.3	11.92 ± 1.9^e^

*F, Firmicutes (p); B, Bacteroidetes (p); T, Tenericutes (p); V, Verrucomicrobia (p); R, Ruminococcaceae (f); L, Lachnospiraceae (f)*.

a *vs. HD, corrected P-value = 0.007*.

b *vs. HD, corrected P-value = 0.04*.

c, d, e *vs. HD, corrected P-value < 0.001*.

f *vs. EAMG vehicle, corrected P-value < 0.001*.

g *vs. EAMG BBmix heat exposed, corrected P-value = 0.02*.

*Statistical significance was assessed by one-way ANOVA test with Dunnett's multiple comparison test*.

## Discussion

Modulation of the gut microbiota and its interactions with the host's immune system by probiotics has been proposed as a novel therapeutic tool for the treatment of autoimmune disorders; altered immunological responses can be skewed and damped by probiotics that exhibit immunomodulatory properties (37–39). Previous reports from Chae's (7) and our (6) groups demonstrated that probiotic administration, according to a prophylactic (7) or preventive (6) treatment protocols, ameliorated EAMG symptoms and modulated AChR-specific immune responses. In this study, we evaluated the probiotic efficacy in the Lewis rat EAMG model, given to animals according to a therapeutic protocol (i.e., treatments started at disease onset). Two lactobacilli (LBmix) and two bifidobacteria (BBmix) strains were administered during EAMG chronic phase of the disease, and a significantly improvement of EAMG was observed in BBmix-treated animals, compared with vehicle- and LBmix-fed groups (Figure 1A), with a parallel reduction of anti-AChR antibody levels (Figure 1C).

Then, we wondered whether the observed clinical efficacy of bifidobacteria in our EAMG model was dependent to their vitality. Indeed, the debate concerning the efficacy of live probiotics compared to inactivated bacteria is still open, with contrasting results. Zimmermann et al. compared the effects of several bacteria strains, live or heat exposed, on a human colorectal adenocarcinoma cell line (HT-29) and observed that live probiotics induced immunosuppressive effects, whereas heat exposed bacteria caused an elevated immune response (40). By contrast, Sugahara et al. observed that both live and heat exposed *B. breve* M-16V suppressed pro-inflammatory cytokine production in spleen cells of gnotobiotic mouse model (41). However, the comparison between the effects of vital or dead cells (irradiated or heat exposed) contained in a mixture of probiotics (VSL3) administered to animals with colitis (DSS-induced colitis), revealed that the non-viable irradiated or viable probiotics attenuated the symptoms, whereas the heat exposed probiotics had no effect on the severity of disease (42). In this regard, results from *in vivo* experiments suggest that the selected bifidobacteria strains, when vital, have a greater efficacy in modulating EAMG compared with vital lactobacilli (Figure 1A) and with heat exposed bifidobacteria (Figure 1B), when given to animals after disease onset (therapeutic protocol), and this effect was associated with reduced serum anti-AChR antibodies titers (Figure 1D). Thus, these data further confirm our previous study on the efficacy of preventive probiotic treatment in the rat EAMG model (6) and show, for the first time, the efficacy of the administration of probiotics to EAMG Lewis rats, following a therapeutic treatment. The beneficial effects of vital bifidobacteria could be related to the maintenance of the integrity of bacterial proteins (e.g., carbohydrate polymers exhibited on the cell surface) and DNA sequences (e.g., CpG motifs), that exhibit immunomodulatory properties (16). The minor effects of heat exposed bifidobacteria could be, therefore, linked to the partial denaturation of molecules with immunomodulatory activity. Moreover, the heat inactivation blocks the metabolite production, that could, even in a small amount, alter the cross-feeding mechanisms among commensal bacteria, important in the regulation of immune system (e.g., short chain fatty acid). However, further studies using different techniques (such as irradiation, formaldehyde, or DNase-treatment) that could inactivate bacteria while retaining the intact protein structures, may provide more insight into the mechanisms by which the probiotics act and into the mediators induced in the EAMG model by our vital or inactive probiotics.

As reported, probiotics are capable to influence the immune system, not only locally but also in the periphery (38). We observed that the therapeutic treatment with BBmix was associated with increased FoxP3 and TGFβ mRNAs expression in drLNs of EAMG animals (Figure 2A). This observation is in line with our previous report on the efficacy of preventive probiotic treatment in this model (6); overall, these data further support the hypothesis of TGFβ as systemic cytokine able to orchestrate the immune-modulation activated by probiotics (6), besides FoxP3^+^cells as key players in probiotic induced-suppressive activity (43, 44).

Whether an interaction of bacteria with GALT could occur has been studied by several researchers and it has been described that DCs in the gut can even send dendrites in the intestinal lumen to sample the microenvironment (10, 45). To study possible interactions of our probiotic strains with immune elements in the gut mucosa, bacterial wall was *in vitro* labeled with WGA-AF555, that selectively binds to N-acetyl-D-glucosamine and sialic-acid containing glycoconjugates and oligosaccharides (46). Super resolution microscopy images showed slightly differences in theWGA-AF555 staining pattern (Figures 3A,B), suggesting a specific distribution of the two WGA ligands in bifidobacteria and in lactobacilli cell wall. Then, WGA-AF555 labeled probiotics were administered to Lewis rats and, after 30–60 min, the intestine were aseptically removed and processed for confocal and super resolution microscopy. Immunofluorescence images demonstrated that WGA-AF555-labeled probiotics were found nearby villi (Figures 3D,E) and in PPs (Figures 3F,G); within PPs, WGA-AF555-labeled probiotics were found in close contact with CD3^+^ cells (Figure 3H) and CD11c^+^ cells (Figure 3I).

Furthermore, by means of *in vitro* live imaging (Figure 4A, Supplementary Video 1) and super resolution microscopy (Figures 4C,D) we were able to confirm that bacterial cells interact BMDCs. These data suggests that DC cells and CD3^+^ lymphocytes could indeed interact with bifidobacteria in PPs; whether these interactions are preliminary to phagocytosis by DCs (47, 48) needs to be further investigated.

DCs make contacts with microbes using pattern recognition receptors (PRRs), which recognize pathogen-associated molecular patterns (PAMPs); among PRRs, toll-like receptors (TLRs) play a pivotal role, and are capable to discriminate different components of bacteria (49). Hence, we evaluated whether the interaction of bifidobacteria with BMDCs could modulate TLRs transcription profile. qRT-PCR analysis showed increased TLR2 transcription in probiotic-treated BMDCs and a weak down-regulation of TLR1 in BBmix-and in lactobacilli-treated BMDCs (Figure 5A); TLR6 mRNA was unaltered. The increased TLR2 mRNA expression was confirmed by confocal (Figures 5B,C) and super resolution microscopy (Figures 5D,E), showing increased TLR2 expression on CD11c^+^ cells and its localization in specific plasma membrane regions. Although BMDC exposure to bifidobacteria and lactobacilli resulted in differential expression of TLR1 and TLR2 mRNAs, as a consequence of cell-cell recognition, their effects on EAMG manifestation were different. The better therapeutic efficacy of bifidobacteria could be partly explained by a differential downstream effect on pathogens recognition pathways by BMDC *in vitro*, possibly in correlation with not yet reported differences on LTA protein structure between bifidobacteria and lactobacilli and their affinity to TLR2. Indeed, we found that lactobacilli strains induce a considerable increase of IL12b mRNA (preliminary data, not shown) besides IL10 mRNA (6) suggesting that lactobacilli could drive the differentiation of naïve T cells toward a Thelper rather than Treg phenotype. All together these findings suggest that TLR2 could be one of the PRRs engaged in the bifidobacteria recognition, while TLR1 and TLR6, functionally associated with TLR2 in the discrimination of a subtle difference between triacyl- and diacyl-lipopeptides (49), are not involved at this stage. Since lipoteichoic acid (LTA), a major cell wall component of gram-positive bacteria, is primarily recognized by TLR2 (50), probiotic exposed-BMDCs were analyzed by immunofluorescence for the co-expression of LTA and TLR2 (Figure 5F), further suggesting that the recognition of our probiotics is mediated by LTA-TLR2 engagement that occurs in TLR2-enriched domains on the BMDC plasma membrane. Further analysis are needed to describe the events occurring in DC cytoplasmic domain below the engaged LTA-TLR2 molecules (51), with regard to the TLR2-signal transduction cascade resulting in the release of immunomodulatory cytokines.

To summarize, results or our research suggest that CD11c^+^ DCs contact probiotics in the GALT (Figure 3) and this interaction is mediated by TLR2 (Figure 4), upregulates immunoregulatory citokines mRNAs (i.e. IL10 and TGFβ) and induces the production of TGFβ (6). Since activated DCs in PPs migrate to mesLN (12), we sought to investigate, using a simplified *in vitro* model of DC-Teffector interactions, a plausible mechanism of immune modulation induced by probiotics *in vivo*. Thus, DCs were exposed to bifidobacteria, and loaded with an antigen involved in EAMG (R97-116). Then, DCs were co-cultured with “circulating” antigen- specific CD4^+^ Teff cells (Figure 6A) and since it has been suggested that probiotics can induce immune tolerance either/or promoting Treg activity and/or suppressing T helper response (5), we wondered whether bifidobacteria treatment could influence Tcell motility, clue of the correct immunological synapse formation (22). Indeed, it has been demonstrated that the formation of a stable major histocompatibility complex (MHC)-peptide-TCR complex between DCs and antigen-specific T-lymphocytes results in a temporary engagement of T cells, characterized by a reduced motility and low meandering index (stationary phenotype), in contrast to not engaged T cells, that remains free to move, with high velocity and elevated meandering index (motile phenotype) (22, 23). Hence, bifidobacteria-exposed and antigen-loaded BMDCs were co-cultured with R97-116 specific CD4^+^ Teff cells (Figure 6A) and, by means of live-imaging microscopy and motility analysis, stationary and motile R97-116 Tcells analyzed. In control co-cultures (i.e., antigen loaded BMDC) more than 70% of Teff had a stationary phenotype (Figure 6C, empty bars); on the contrary, when Teff cells were co-cultured with BBmix-exposed BMDCs, the majority of tracked cells displayed a motile phenotype (Figure 6B, black bars), and an increased mean velocity (Figure 6D, black bars), suggesting that antigen-specific T lymphocytes continued to patrol the microenvironment near BMDCs, presumably still searching the engagement with the proper MHC-peptide complex. Whether this different Teff behavior is due to a reduced density of MHC-peptide complexes or of co-stimulatory molecules on BMDCs, is a point that deserves further studies.

In our previous study (6), we demonstrated that TGFβ was involved in the immune-modulation observed in probiotic treated EAMG rats (preventive protocol); of note, TGFβ was found increased in drLNs from BBmix-vital treated EAMG rats (therapeutic protocol) (Figure 2A). Since the role of TGFβ in association to tolerance induction in EAMG has been reported in several studies (52, 53), we choose to expose BMDCs to TGFβ prior to the antigen-loading step and co-culture with R97-116 specific CD4^+^ Teff cells; then motility analyses were performed (Figures 6B–G, striped bars). Our data showed that TGFβ is able to reproduce the effects observed with BBmix exposed BMDCs, i.e., increased percentages of motile Teff (Figure 6B, striped bars), characterized by a greater mean velocity (Figure 6C, striped bars). Overall, these data show that the exposition of BMDCs to bifidobacteria cause a downstream effect on Tcell motility, mimicking the same results obtained with the TGFβ. Hence, we can hypothesized that BBmix treatment could lead to a T cell phenotype more tolerant, resembling the effects induced by TGFβ in DC *in vitro* (i.e., downregulation of antigen-presenting function and expression of co-stimulatory molecules) (52). The exact molecular mechanism that lead toward a tolerant phenotype needs to be elucidated.

The effects of probiotics on the host immune system could be also mediated by a modulation of the gut microbiota; indeed, several studies reported alterations in bacterial taxonomical composition in stools from patients and from animal models (54, 55), suggesting an important role of microbiota in (auto)immune diseases. To our knowledge, this is the first study that characterize the intestinal microbiota composition in the Lewis rat EAMG model, considering also the effect of a probiotic therapeutic treatment. The analysis of the gut microbiota demonstrated differences between HD and EAMG rats, and during progression of EAMG from onset to the chronic phase (end of experiments) (Figures 7A–C), with an higher microbial diversity at the onset stage of the disease (Figure 8D).

At the *phylum* level, *Firmicutes* and *Bacteroidetes* represent almost the totality of the microbial population both in healthy and EAMG rats (Figure 8A), and the *Firmicutes*/*Bacteroidetes* (F/B) ratio is used to describe a pro-inflammatory microbiota (56–59); however, in the different experimental conditions of our study, the F/B ratio was unaltered (Table 1), suggesting the absence of pathological disequilibrium in the gut microbiota (dysbiosis). The microbial alteration observed during EAMG course mostly regarded the less abundant bacterial populations at each taxonomic level (Figures 8B,D). Overall, we observed an increased abundance of certain families/genera in EAMG onset compared to the other experimental groups (Table 1), and that the probiotic treatment is associated with modulation of the relative abundance of certain microbial community (Supplementary Figure 3). Of note, this disequilibrium seems to be partially reverted in BBmix vital EAMG rats, as it also occurs in the last phase of the disease (EAMG chronic). The most affected bacterial populations were *Tenericutes* and *Verrucomicrobia* phyla, *Lachnospiraceae* family, *Turicibacter, Lactobacillus*, and *Akkermansia* genera. The decrease of *Tenericutes* phylum was also observed in intestinal dysbiosis of rats due to inflammatory conditions (60), as well as the increase of *Verrucomicrobia* (61). Moreover, it has been reported a positive correlation of members belonging to the *Lachnospiraceae* family as immune-modulating bacteria in rats affected by experimental autoimmune encephalomyelitis (62). Thus, the decreased levels of *Lachnospiraceae* in the EAMG chronic group could be related to the inflammatory status, whereas the treatment with BBmix vital could contribute to its increase. This effect was also observed in gut microbiota of patients with inflammatory bowel disease (63). We observed the increase of *Akkermansia* in rats in the chronic phase of the disease compared to both healthy rats and rats treated with bifidobactiera. It is known that *Akkermansia muciniphila*, belonging to the genera *Akkermansia*, is a mucin-degrader bacterium and it has been reported to have both regulatory and inflammatory properties (64). The increase of this genus has been reported in other autoimmune pathologies, such as multiple sclerosis (64, 65) and type 1 diabetes patients (66). The reasons of this increase can be found in its dual functions: it can increase to compensate for the imbalance in gut microbiota composition or it can contribute itself to the disequilibrium. However, a deepened analysis of the genus *Akkermansia* and a more complex evaluation of bacteria and their metabolites are required to understand the EAMG gut microbiota profile. We were unable to detect changes in the relative abundance of Bifidobacterium genera by 16S rRNA NGS sequencing in gut microbiota; this could be explained by the fact that NGS approach is not truly quantitative, and more specific and sensitive methods (e.g., strain specific RT-qPCR assays) should be employed (6).

Our data suggest that an ongoing immune sensitization process, from EAMG induction to EAMG onset, could contribute to the microbiota imbalance that could be counteracted during the disease course. Furthermore, microbiota analysis suggests that, even if an evident alteration of gut microbiota can be observed at EAMG onset, the administration of bifidobacteria, either vital or heat exposed, could help in restoring the normal gut microbiota balance, as observed in HD animals. However, we cannot exclude that the systemic activation of the immune system, as a consequence of the active TAChR/CFA immunization, could influence the GALT thus playing a crucial, although indirect, role in the gut microbial alterations. Administration of probiotics is not associated to major modification in the most abundant microbiota phyla present in EAMG animals, and more focused studies are necessary to specifically target the bifidobacterium genus, and particularly in relation to the probiotic administration. Moreover, the beneficial effects associated to probiotic administration still deserve proper investigation, due to heterogeneity of metabolic mediators, such as short chain fatty acid (67), produced by the gut flora and involved in the crosstalk between microbiota and the immune system.

In conclusion, our study demonstrated that the therapeutic administration of two bifidobacteria probiotic strains induced immunomodulatory effects leading to EAMG amelioration. Furthermore, inactivated probiotic by heat exposure were less effective; *in vitro* experiments demonstrate that LTA-TLR2 interaction does occur between probiotics and DCs, and TLR2 undergoes a membrane redistribution that could interfere with the formation of the MHC-Ag-TCR complex between DCs and AChR specific T cells; BMDCs exposed to TGFβ alters Tcells motility pattern similar to that observed by bifidobacteria exposed BMDCs. Finally, EAMG induction is associated to an altered gut microbiota, and probiotic intake could contribute to restore the normal microbiota.

In MG, innovative therapies counteracting the altered autoimmune attack and loss of tolerance to AChR, possibly without side effects, are still needed. To reach this aim, the selection and characterization of probiotic strains with immunomodulatory properties could be of relevance for MG and other autoimmune diseases.

## Data Availability Statement

The raw data concerning metagenomic analysis and supporting the conclusions of this manuscript will be made available by the authors, without undue reservation, to any qualified researcher.

## Ethics Statement

The animal study was reviewed and approved by the Institute Ethical Board and the Italian Ministry of Health (code 1064/2015-PR).

## Author Contributions

ER, AC, CCo, GS, and CCr generated the data. ER, AC, AF, CCo, and FB analyzed the data. ER, AC, AF, and FB wrote the manuscript. All authors approved the final manuscript.

### Conflict of Interest

ME was employed by company AAT—Advanced Analytical Technologies. The remaining authors declare that the research was conducted in the absence of any commercial or financial relationships that could be construed as a potential conflict of interest.
