# Supplementary material for: Therapeutic Effect of Bifidobacterium Administration on Experimental Autoimmune Myasthenia Gravis in Lewis Rats
Source: Front Immunol. 2019 Dec 19;10:2949. doi: 10.3389/fimmu.2019.02949 (PMC6951413; doi:10.3389/fimmu.2019.02949)
Supplement: Supplementary file 5 [file Data_Sheet_1.docx]

Supplementary Material





**Supplementary Figure S1**: Body weight (mean±SD) of EAMG rats treated with BBmix, LBmix or vehicle (A) and EAMG rats treated with BBmix vital, BBmix heat exposed or vehicle (B). One-way ANOVA test with Dunnett’s multiple comparison test was used. Corrected p values are reported.





Supplementary Figure S2: RT-qPCR analysis of CHRNA1, Rapsyn and LRP4 mRNAs in muscle of healthy rats and EAMG rats treated with vehicle, BBmix vital or BBmix heat exposed. Statistical significance was assessed by one-way ANOVA test with Dunnett’s multiple comparison test.


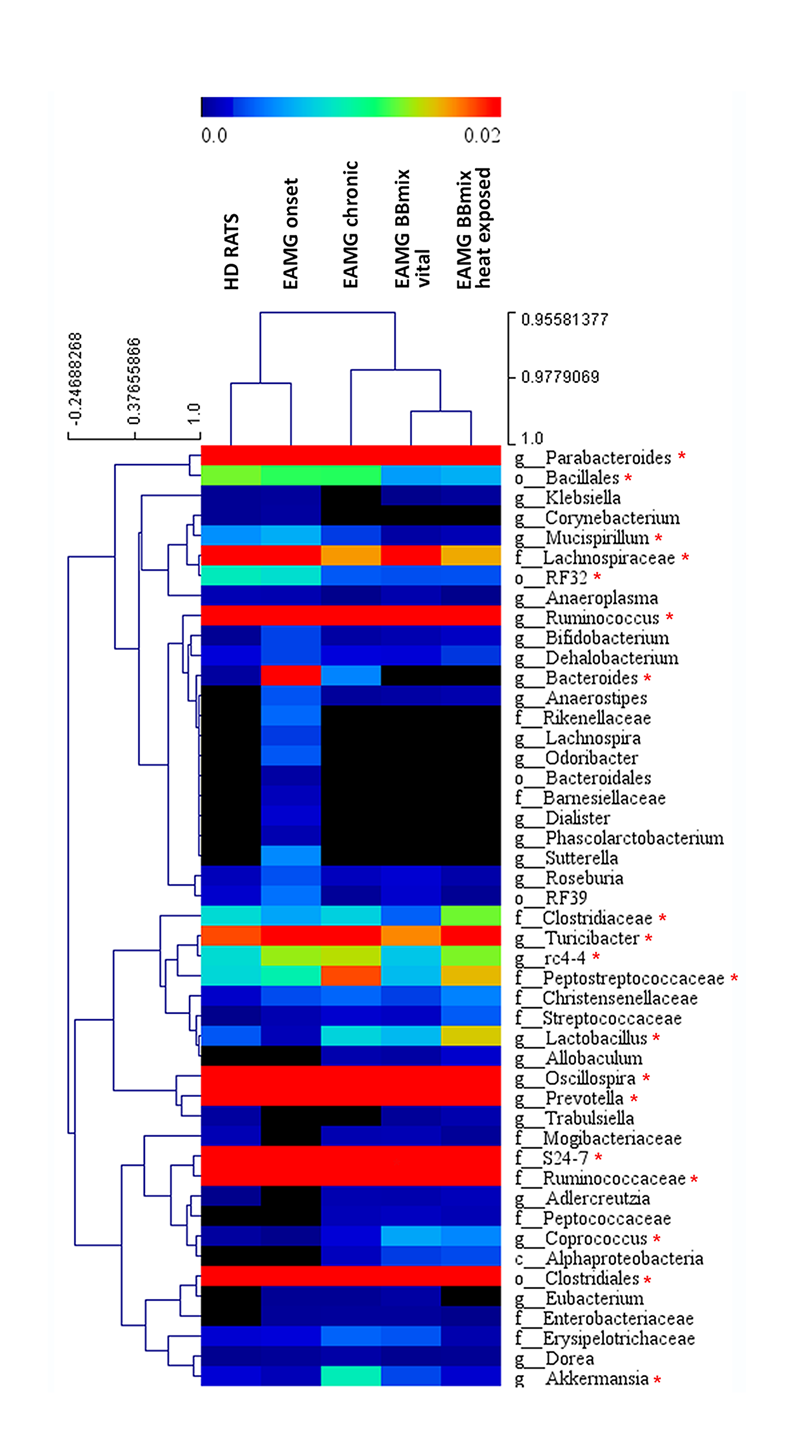


**Supplementary Figure S3:** The heatmap plot describes the relative abundance (taxa accounting for >0.1% are reported). of each bacterial genus (rows) within each sample (column). The color code (blue to red) displays the row z-score: red color indicates high abundance, blue color low abundance. The dendrogram shows hierarchical clustering of bacterial communities based on the Pearson correlation coefficient as the measure of similarity. Letters preceding taxonomic labels indicate the taxonomic level (o=order, f=family, g=genus). Genera with relative abundance higher than 1% are highlighted with a red star.

**Supplementary Video 1:** *In vitro* co-culture of BMDCs with WGA-AF555 labelled *Lactobacillus rhamnosus* (LR). Overlaid images of red fluorescence and differential interference contrast channels. Observation time: 60 minutes. 30 seconds intervals. Objective 60X.

**Supplementary Video 2:** Time-lapse video-microscopy of rat CFSE labelled-R97-116 Teff (green) in co-culture with rat R97-116-loaded BMDCs. Overlaid images of green fluorescence and differential interference contrast channels. Observation time: 60 minutes. 30 seconds intervals. Objective 20X.

**Supplementary Video 3:** Time-lapse video-microscopy of rat CFSE labelled-R97-116 Teff (green) in co-culture with rat R97-116-loaded BMDCs pre-incubated with BBmix. Overlaid images of green fluorescence and differential interference contrast channels. Observation time: 60 minutes. 30 seconds intervals. Objective 20X.

**Supplementary Video 4:** Time-lapse video-microscopy of rat CFSE labelled-R97-116 Teff (green) in co-culture with R97-116-loaded BMDCs pre-incubated with TGFβ. Overlaid images of green fluorescence and differential interference contrast channels. Observation time: 60 minutes. 30 seconds intervals. Objective 20X.
